# Peroxisome proliferator-activated receptor alpha acts as a mediator of endoplasmic reticulum stress-induced hepatocyte apoptosis in acute liver failure

**DOI:** 10.1242/dmm.023242

**Published:** 2016-07-01

**Authors:** Li Zhang, Feng Ren, Xiangying Zhang, Xinxin Wang, Hongbo Shi, Li Zhou, Sujun Zheng, Yu Chen, Dexi Chen, Liying Li, Caiyan Zhao, Zhongping Duan

**Affiliations:** 1 Department of Infectious Diseases, The Third Affiliated Hospital of Hebei Medical University, Shijiazhuang 050051, China; 2 Beijing Artificial Liver Treatment and Training Center, Beijing YouAn Hospital, Capital Medical University, Beijing 100069, China; 3 Beijing Institute of Hepatology, Beijing YouAn Hospital, Capital Medical University, Beijing 100069, China; 4 Department of Pathology, Beijing YouAn Hospital, Capital Medical University, Beijing 100069, China; 5 Department of Cell Biology, Municipal Laboratory for Liver Protection and Regulation of Regeneration, Capital Medical University, Beijing 100069, China

**Keywords:** Peroxisome proliferator-activated receptor α, Endoplasmic reticulum stress, Acute liver failure, Hepatotoxicity, Apoptosis

## Abstract

Peroxisome proliferator-activated receptor α (PPARα) is a key regulator to ameliorate liver injury in cases of acute liver failure (ALF). However, its regulatory mechanisms remain largely undetermined. Endoplasmic reticulum stress (ER stress) plays an important role in a number of liver diseases. This study aimed to investigate whether PPARα activation inhibits ER stress-induced hepatocyte apoptosis, thereby protecting against ALF. In a murine model of D-galactosamine (D-GalN)- and lipopolysaccharide (LPS)-induced ALF, Wy-14643 was administered to activate PPARα, and 4-phenylbutyric acid (4-PBA) was administered to attenuate ER stress. PPARα activation ameliorated liver injury, because pre-administration of its specific inducer, Wy-14643, reduced the serum aminotransferase levels and preserved liver architecture compared with that of controls. The protective effect of PPARα activation resulted from the suppression of ER stress-induced hepatocyte apoptosis. Indeed, (1) PPARα activation decreased the expression of glucose-regulated protein 78 (Grp78), Grp94 and C/EBP-homologous protein (CHOP) *in vivo*; (2) the liver protection by 4-PBA resulted from the induction of PPARα expression, as 4-PBA pre-treatment promoted upregulation of PPARα, and inhibition of PPARα by small interfering RNA (siRNA) treatment reversed liver protection and increased hepatocyte apoptosis; (3) *in vitro* PPARα activation by Wy-14643 decreased hepatocyte apoptosis induced by severe ER stress, and PPARα inhibition by siRNA treatment decreased the hepatocyte survival induced by mild ER stress. Here, we demonstrate that PPARα activation contributes to liver protection and decreases hepatocyte apoptosis in ALF, particularly through regulating ER stress. Therefore, targeting PPARα could be a potential therapeutic strategy to ameliorate ALF.

## Abstract (precis)

**Summary:** Upregulation of PPARα can ameliorate hepatic injury by inhibiting ER stress-mediated hepatocyte apoptosis in a mouse model of D-GalN/LPS-induced acute liver failure.

## INTRODUCTION

Acute liver failure (ALF) is a clinical syndrome defined by the sudden onset of severe liver injury and is characterized by encephalopathy and coagulopathy in individuals with previously normal liver function (Khan et al., 2006). The causes of ALF are diverse, including toxins, infections or metabolic and genetic diseases, but irrespective of etiology, ALF results from rapid and extensive hepatic apoptosis and necrosis (Riordan and Williams, 2003). Despite developments in treatment, orthotopic liver transplantation (OLT) is still considered the most effective therapy. Unfortunately, the feasibility of OLT is extremely limited by the rapid progression of the disease and the shortage of donor livers; therefore, the pathogenesis of ALF needs to be further explored.

Peroxisome proliferator-activated receptors (PPARs) are members of the nuclear hormone receptor superfamily of ligand-inducible transcription factors. To date, three subtypes of PPARs (α, β, γ) have been identified in many species, including humans (Desvergne and Wahli, 1999; Kota et al., 2005). PPARα has been reported to regulate lipid metabolism (Staels et al., 1998), inflammation (Devchand et al., 1996; Delerive et al., 1999), cell differentiation and apoptosis (Roberts et al., 2002). Studies have demonstrated that PPARα plays a different role in cancer cells than in normal cells. PPARα activation is commonly implicated in hepatocarcinogenesis protocols for rodents in which its anti-apoptotic action is assumed to play a crucial role (Misra et al., 2013; Misra and Reddy, 2014); however, activation of PPARα by exogenous agonists reduces tumor cell growth in cell lines derived from colorectal cancer (Grau et al., 2006). In non-cancerous renal tubular cells, a lack of PPARα exacerbates gentamicin-induced apoptosis (Hsu et al., 2008). Additionally, Wy-14643, a potent exogenous PPARα ligand and a selective PPARα agonist (Cuzzocrea et al., 2004; Briguglio et al., 2010), decreases the apoptosis of cardiomyocytes via reducing the nuclear translocation of nuclear factor-κB (NF-κB) and reducing caspase-3 activation, thus preserving myocardial function and maintaining cardiac contractility (Yeh et al., 2006). In a third normal cell system, PPARα agonist treatment has been shown to increase trefoil factor family-3 expression and attenuate apoptosis in the liver tissue of bile duct-ligated rats (Karakan et al., 2013). Our recent study has shown that PPARα activation protects the liver from acute injury by promoting the autophagy pathway in the D-galactosamine (D-GalN)- and lipopolysaccharide (LPS)-induced ALF mouse model (Jiao et al., 2014). However, whether PPARα plays a protective role in the liver by inhibiting hepatocyte apoptosis is yet to be determined.

The endoplasmic reticulum (ER) is a vital cellular organelle for the protein folding and trafficking, lipid synthesis and calcium homeostasis that are required for cell survival and functions. Endoplasmic reticulum stress (ER stress) is induced by physiological and/or pathological stress signals, leading to the accumulation of unfolded or misfolded proteins in the ER, and activates three ER-localized transmembrane protein sensors (Ron and Walter, 2007; Lin et al., 2008). The chaperone proteins glucose-regulated protein 78 (Grp78) and glucose-regulated protein 94 (Grp94) are master regulators of ER homeostasis and are hallmarks for ER stress responses (Little et al., 1994). The coordinated adaptive response is known as the unfolded protein response (UPR), and the pathological response is known as the ER stress response. The UPR signaling pathways act rapidly to mitigate the stressed state of the ER and enhance cell survival. However, if severe and prolonged ER stress cannot be resolved, the signaling switches from a pro-survival to a pro-apoptotic ER stress response (Xu et al., 2005). Compelling evidence has suggested that C/EBP-homologous protein [CHOP, also known as growth arrest and DNA damage-inducible protein 153 (GADD153)] and caspase-12 in rodents (caspase-4 in humans) are activated and become involved in ER stress-induced cell apoptosis (Kim et al., 2008). As reported previously, PPARα protects HepG2 cells against H_2_O_2_-induced ER stress-mediated apoptosis through the downregulation of CHOP (Tang et al., 2014). Additionally, activation of PPARα ameliorates hepatic insulin resistance to increased ER stress (Chan et al., 2013). However, a PPARα agonist has also been shown to induce apoptosis of triple-negative breast cancer cells via activation of the transcription factor NF-κB, which is connected with the ER stress response (Zhao et al., 2007). Thus, these studies have demonstrated that PPARα plays a complicated role in ER stress.

Although our studies have demonstrated that PPARα activation effectively protects mice from ALF, and severe ER stress promotes liver injury by inducing hepatocyte apoptosis in D-GalN/LPS-treated mice (Jiao et al., 2014; Ren et al., 2015), the underlying mechanisms of the effects of PPARα and ER stress *in vivo* required further elucidation. Thus, this study sought to address the hypothesis that PPARα can protect mice from ALF by inhibiting ER stress-induced hepatocyte apoptosis. Indeed, we found that inhibition of ER stress enhanced the expression of PPARα, and PPARα activation attenuated ER stress-mediated hepatocyte apoptosis in the D-GalN/LPS-induced mouse model of ALF.

## RESULTS

### PPARα activation decreases hepatocyte apoptosis, thus protecting against ALF

**Fig. 1.**
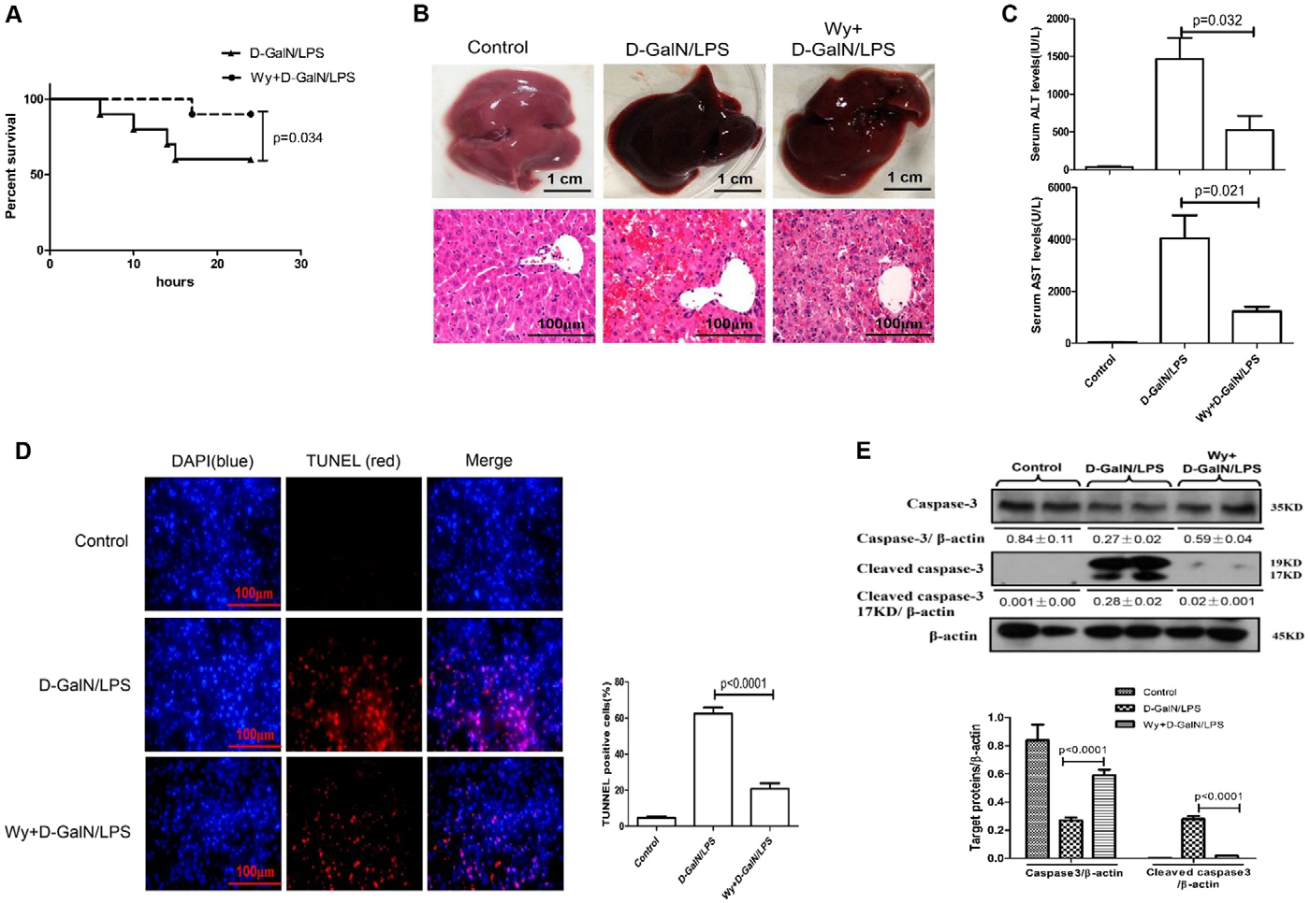
**Wy-14643 protects against D-GalN/LPS-induced liver injury and suppresses hepatocyte apoptosis.** Male C57BL/6 mice were injected intraperitoneally with Wy-14643 (6 mg/kg) or vehicle (DMSO) 2 h prior to D-GalN (700 mg/kg) and LPS (10 μg/kg) exposure (*n*=12/group). The control mice were pre-treated with vehicle (DMSO) 2 h before PBS injection (*n*=10). One group of mice were euthanized with chloral hydrate (1.0 g/kg) 6 h after D-GalN/LPS treatment, and the liver and serum samples were collected for analysis. (A) In a second group of mice, the survival rate was analyzed in D-GalN/LPS-treated mice and Wy/D-GalN/LPS-treated mice up to 24 h after D-GalN/LPS injection. (*n*=10/group). (B) Representative livers and H&E staining of liver sections in control mice, D-GalN/LPS-treated mice, and Wy/D-GalN/LPS-treated mice. (C) Serum levels of ALT and AST from the three treatment groups. (D) TUNEL staining images from the three treatment groups. A representative experiment is shown. Original magnification 200×. (E) The levels of total caspase-3, cleaved caspase-3 and β-actin were measured by western blotting for the three treatment groups. A representative blot from two samples of every group is shown. Densitometry analysis of protein levels was performed for each sample. Data in C-E represented as means±s.d.

We first evaluated whether PPARα activation could rescue liver injury by applying Wy-14643, a PPARα ligand activator. In the survival analysis (Fig. 1A), the mice in the D-GalN/LPS group began to die 6 h after D-GalN/LPS administration, and the survival rate stabilized at 60% (6 of 10 mice) at 24 h; however, pre-treatment with Wy-14643 before D-GalN/LPS administration reduced the mortality, and the survival rate was 90% (9 of 10 mice). With respect to liver damage, compared with the D-GalN/LPS administration group, the gross morphology of the liver seemed to be substantially better and the liver histopathological damages were ameliorated in the Wy-14643 treatment group (Fig. 1B). Liver function showed significantly lower alanine aminotransferase (ALT) and aspartic aminotransferase (AST) levels and lower total bilirubin (TBIL), alkaline phosphatase (ALP) and prothrombin time (PT) in the Wy-14643 pre-treatment group compared with the D-GalN/LPS administration group (Fig. 1C; Fig. S1, Table S2). To explore the potential protective mechanism of PPARα against ALF induced by D-GalN/LPS, we measured apoptotic cells in the three groups. As shown in Fig. 1D, in the D-GalN/LPS-treated group, a large number of TUNEL-positive cells were observed; however, the Wy-14643 pre-treatment group displayed significantly fewer apoptotic hepatocytes. Moreover, consistently with the TUNEL data, the levels of cleaved caspase-3 (17 and 19 kDa) increased after D-GalN/LPS injection, but this increase was attenuated by Wy-14643 pre-treatment (Fig. 1E). Thus, these results suggest that PPARα activation significantly reduces apoptotic cells and thereby protects mice from ALF induced by D-GalN/LPS.

### PPARα activation relieves ER stress in D-GalN/LPS-induced ALF

**Fig. 2.**
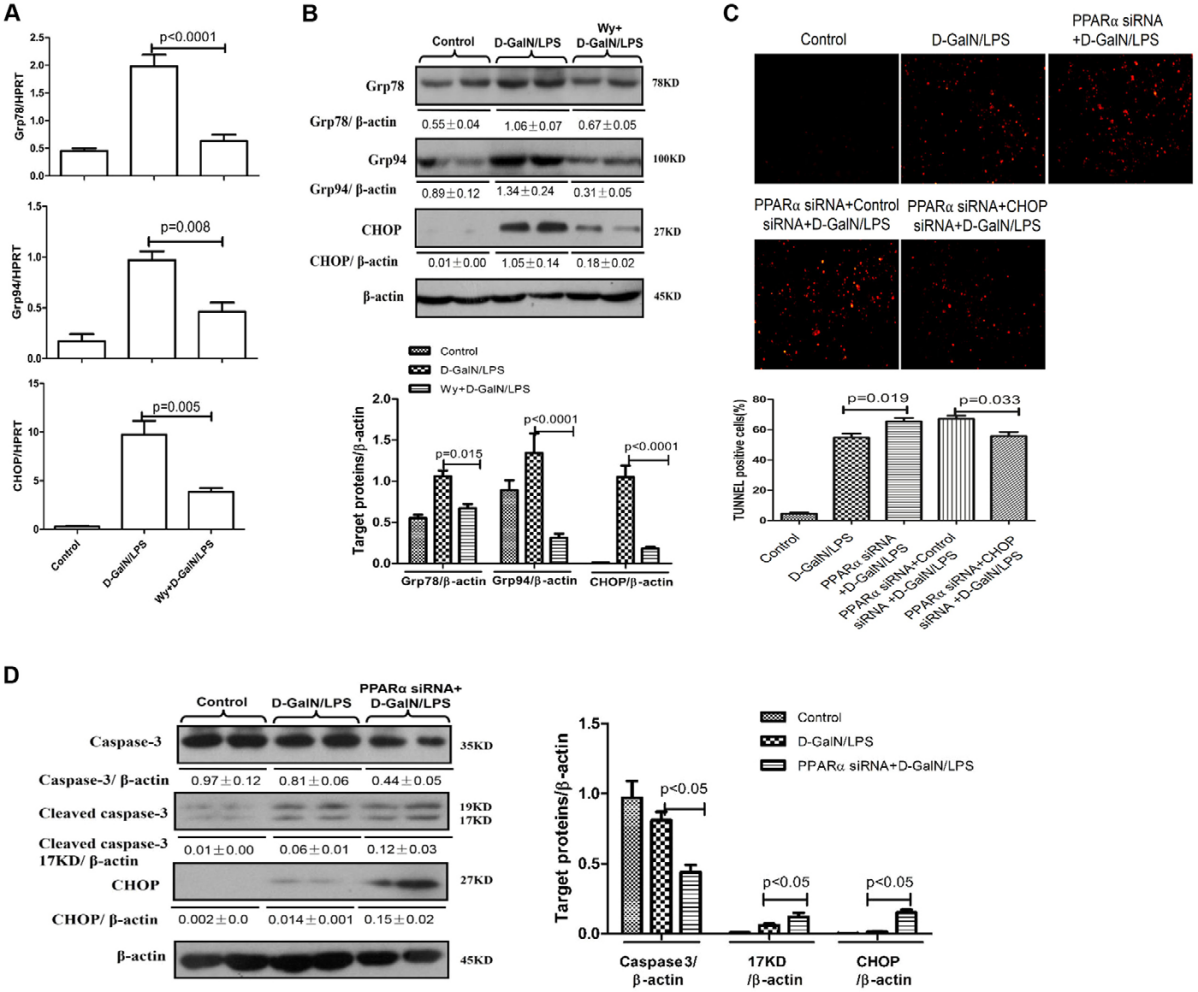
**Wy-14643 suppresses ER stress in D-GalN/LPS-induced ALF.** Male C57BL/6 mice were injected with Wy-14643 (6 mg/kg) or DMSO 2 h prior to D-GalN (700 mg/kg) and LPS (10 μg/kg) treatment (*n*=12/group). In C and D, mice were pre-treated with PPARα siRNA (50 μM/kg) and/or CHOP siRNA (50 μM/kg) via tail vein injection 24 h prior to D-GalN/LPS treatment (*n*=10/group). The control mice were injected with only PBS (*n*=10). The mice were euthanized 6 h after D-GalN/LPS treatment, and the liver and serum samples were collected. (A) Relative hepatic mRNA expression levels of ER stress markers, including Grp78, Grp94 and CHOP were measured by qRT-PCR in the control mice, the D-GalN/LPS-treated mice, and the Wy/D-GalN/LPS-treated mice. (B) Protein levels of Grp78, Grp94, CHOP and β-actin were measured by western blotting. A representative blot from two samples of every group is shown. Densitometry analysis of protein levels was performed for each sample. (C) TUNEL staining images from control mice, D-GalN/LPS-treated mice, PPARα siRNA/D-GalN/LPS-treated mice, PPARα siRNA/control siRNA/D-GalN/LPS-treated mice and PPARα siRNA/CHOP siRNA/D-GalN/LPS-treated mice. A representative experiment is shown. Original magnification 200×. (D) The levels of total caspase-3, cleaved caspase-3, CHOP and β-actin in control, D-GalN/LPS-treated mice and PPARα siRNA/D-GalN/LPS-treated mice were measured by western blotting. A representative blot from two samples of every group is shown. Densitometry analysis of protein levels was performed for each sample. Data represented as means±s.d.

Our previous paper has shown that severe ER stress promotes liver injury in the D-GalN/LPS-induced ALF mouse model (Ren et al., 2015). To examine the effects of PPARα on D-GalN/LPS-induced ER stress in mice, we measured the levels of mRNA and protein for ER stress mediators. The expression of Grp78, Grp94 and CHOP, which are the classical ER stress markers, was increased significantly after D-GalN/LPS administration but was significantly attenuated by pre-treatment with Wy-14643 (Fig. 2A). These alterations were confirmed by western blot analysis (Fig. 2B). We also used siRNA to knock down the expression of PPARα in mice and found that, compared with D-GalN/LPS-treatment, PPARα siRNA treatment further increased the levels of hepatocyte apoptosis (TUNEL) and promoted the cleavage of caspase-3 and the expression of CHOP in D-GalN/LPS-treated ALF mice (Fig. 2C,D). Additionally, we further used siRNA to knock down CHOP and analyse the hepatocyte apoptosis of liver. The results showed that, compared with the mice pre-treated by PPARα siRNA, the intervention of CHOP siRNA decreased again the number of hepatocyte apoptosis (Fig. 2C). The results showed that PPARα activation significantly decreased ER stress during D-GalN/LPS-induced ALF.

### Inhibition of ER stress increases the expression of PPARα in D-GalN/LPS-induced ALF

**Fig. 3.**
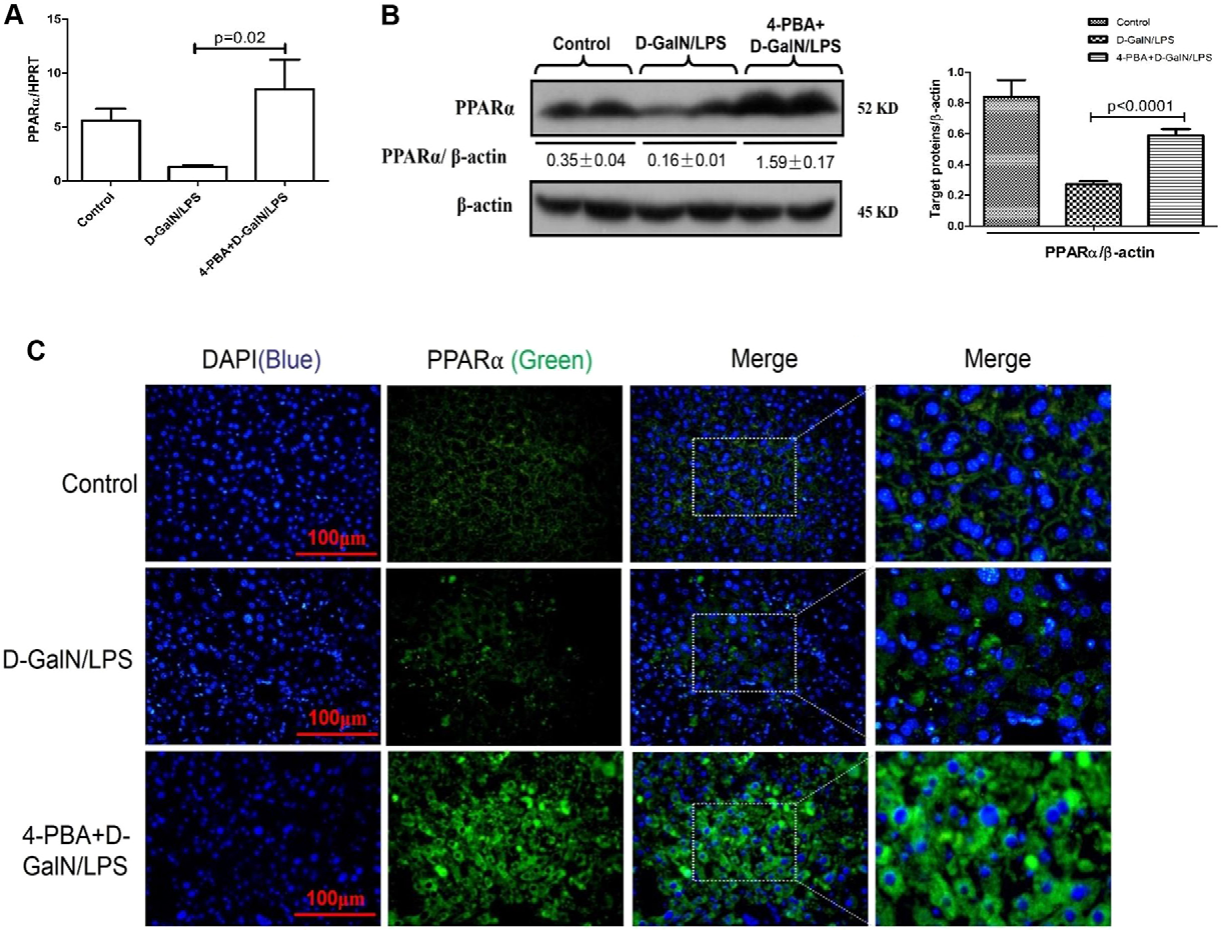
**Inhibition of the ER stress increases the expression of PPARα in D-GalN/LPS-induced ALF.** Mice were pre-treated with 4-PBA (100 mg/kg) or PBS by intraperitoneal injection 6 h prior to the D-GalN (700 mg/kg) and LPS (10 μg/kg) treatment (*n*=14/group). The control mice were injected with only PBS (*n*=10). All mice were finally euthanized with chloral hydrate (1.0 g/kg) 6 h after D-GalN/LPS injection. (A) Relative hepatic PPARα mRNA expression was measured by qRT-PCR in control mice, D-GalN/LPS-treated mice and 4-PBA/D-GalN/LPS-treated mice. (B) Protein levels of PPARα and β-actin were measured by western blotting. A representative blot from two samples of the three treatment groups is shown. Densitometry analysis of protein levels was performed for each sample. (C) Immunofluorescence staining for PPARα (green) in liver tissues from the three treatment groups. A representative experiment is shown. Original magnification 400×. Data in A,B represented as means±s.d.

A small chemical chaperone, 4-phenylbutyric acid (4-PBA), has been shown to alleviate ER stress both *in vivo* and *in vitro* (Özcan et al., 2006; Zode et al., 2011), and inhibition of ER stress by 4-PBA protects mice from ALF induced by D-GalN/LPS (Ren et al., 2015). Thus, we evaluated whether ER stress inhibition could promote the expression of PPARα in the context of ALF. qRT-PCR and western blotting results showed that, compared with D-GalN/LPS treatment alone, pre-treatment with 4-PBA promoted the expression of PPARα (Fig. 3A,B). Similar results were obtained by immunofluorescence staining of liver tissue. Moreover, our results also showed that the expression of PPARα was cytoplasmic rather than nuclear in the three groups (Fig. 3C). These results indicated that the expression of PPARα is promoted by 4-PBA pre-treatment in D-GalN/LPS-induced ALF.

### Inhibition of ER stress protects mice from ALF through PPARα mechanisms

**Fig. 4.**
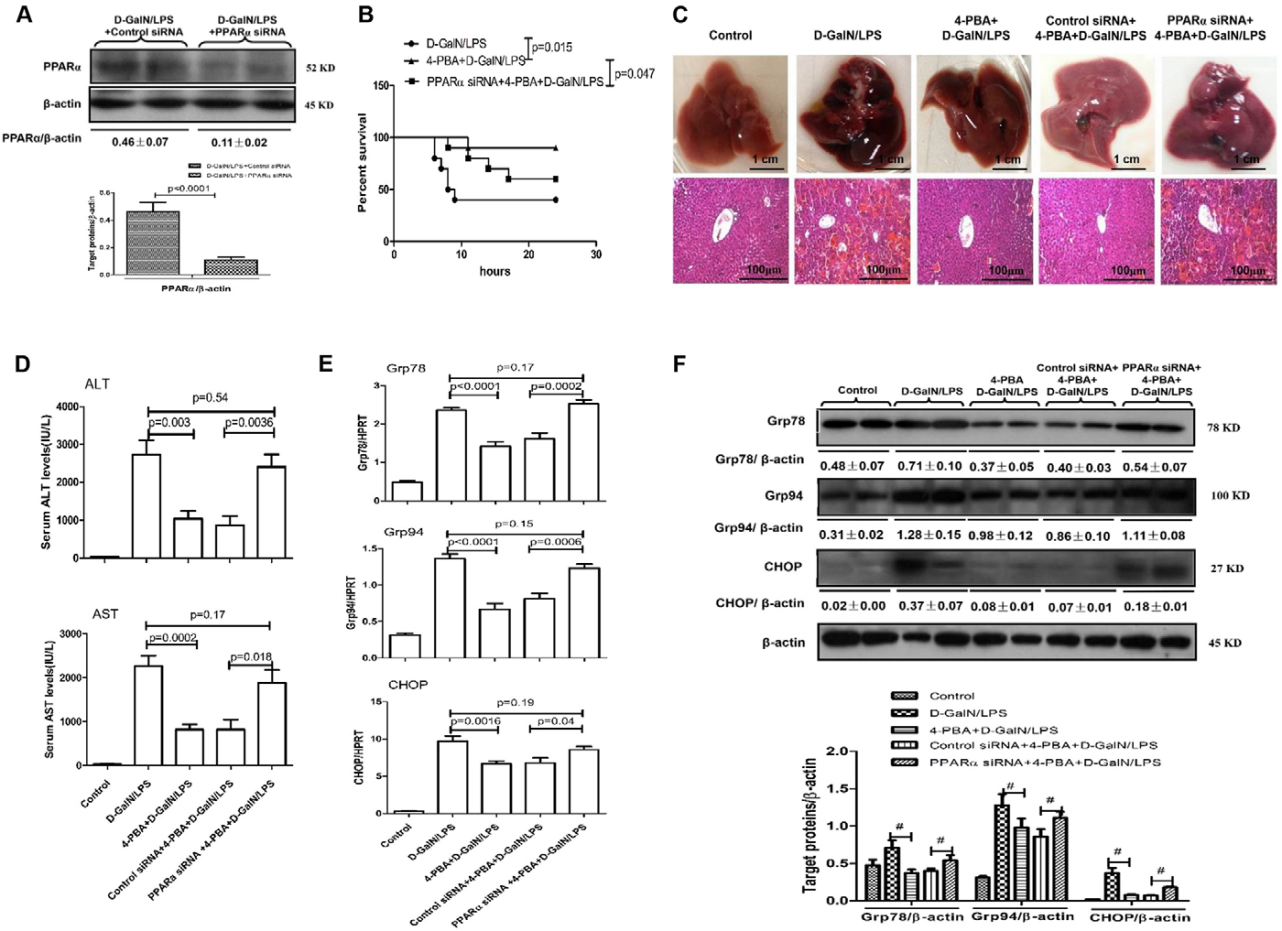
**4-PBA protects against D-GalN/LPS-induced ALF in mice by promoting PPARα activation.** Mice were pre-treated with PPARα siRNA (50 μM/kg) or control siRNA (50 μM/kg) via tail vein injection and then further injected with 4-PBA (100 mg/kg) or PBS, 24 h and 6 h prior to D-GalN (700 mg/kg) and LPS (10 μg/kg) treatment (*n*=14/group), respectively. The control mice were injected with only PBS (*n*=10). One group of mice were euthanized 6 h after D-GalN/LPS treatment, and the liver and serum samples were collected. (A) Protein levels of PPARα and β-actin were measured by western blotting in the control siRNA/D-GalN/LPS-treated mice and the PPARα siRNA/D-GalN/LPS-treated mice. A representative blot from two samples of every group is shown. Densitometry analysis of protein levels was performed for each sample. (B) In a second group of mice, the survival rate was analyzed in D-GalN/LPS-treated mice, 4-PBA/D-GalN/LPS-treated mice and PPARα siRNA/4-PBA/D-GalN/LPS-treated mice up to 24 h after D-GalN/LPS injection. (*n*=10/group). (C) Representative livers and H&E staining of liver sections from control mice, D-GalN/LPS-treated mice, 4-PBA/D-GalN/LPS-treated mice, control siRNA/4-PBA/D-GalN/LPS-treated mice, and PPARα siRNA/4-PBA/D-GalN/LPS-treated mice. (D) Serum levels of ALT and AST from the five treatment groups described in C. (E) Relative hepatic mRNA expression levels of ER stress markers Grp78, Grp94 and CHOP in the five treatment groups were measured by qRT-PCR. (F) Protein levels of Grp78, Grp94, CHOP and β-actin in the five treatment groups were measured by western blotting. A representative blot from two samples of every group is shown. Densitometry analysis of protein levels was performed for each sample (^#^*P*<0.05). Data in A,D-F represented as means±s.d.

Next, we sought to confirm whether the inhibition of ER stress protects the liver from injury by inducing PPARα expression in mice. We used siRNA to knock down the expression of PPARα in mice. The specific inhibition of PPARα in the liver by siRNA *in vivo* was conﬁrmed by the reduced levels of PPARα in mice (Fig. 4A). The results indicated that liver in mice receiving 4-PBA treatment suffered less liver injury, and this hepatic protection was abolished by knockdown of PPARα, which was evidenced by the decreased survival rate (Fig. 4B), abnormal gross morphology and less preserved liver architecture as observed from histology (Fig. 4C) and the significantly higher levels of ALT, AST, TBIL and ALP (Fig. 4D; Fig. S1, Table S2). Conversely, the knockdown of PPARα reversed the expression levels of Grp78, Grp94 and CHOP in 4-PBA-pre-treatment ALF mice (Fig. 4E,F). Thus, these results demonstrate that the mechanism of hepatoprotection by ER stress inhibition depends on PPARα activity.

### The expression profile of PPARα in the progression of ER stress-induced hepatocyte apoptosis *in vitro*

**Fig. 5.**
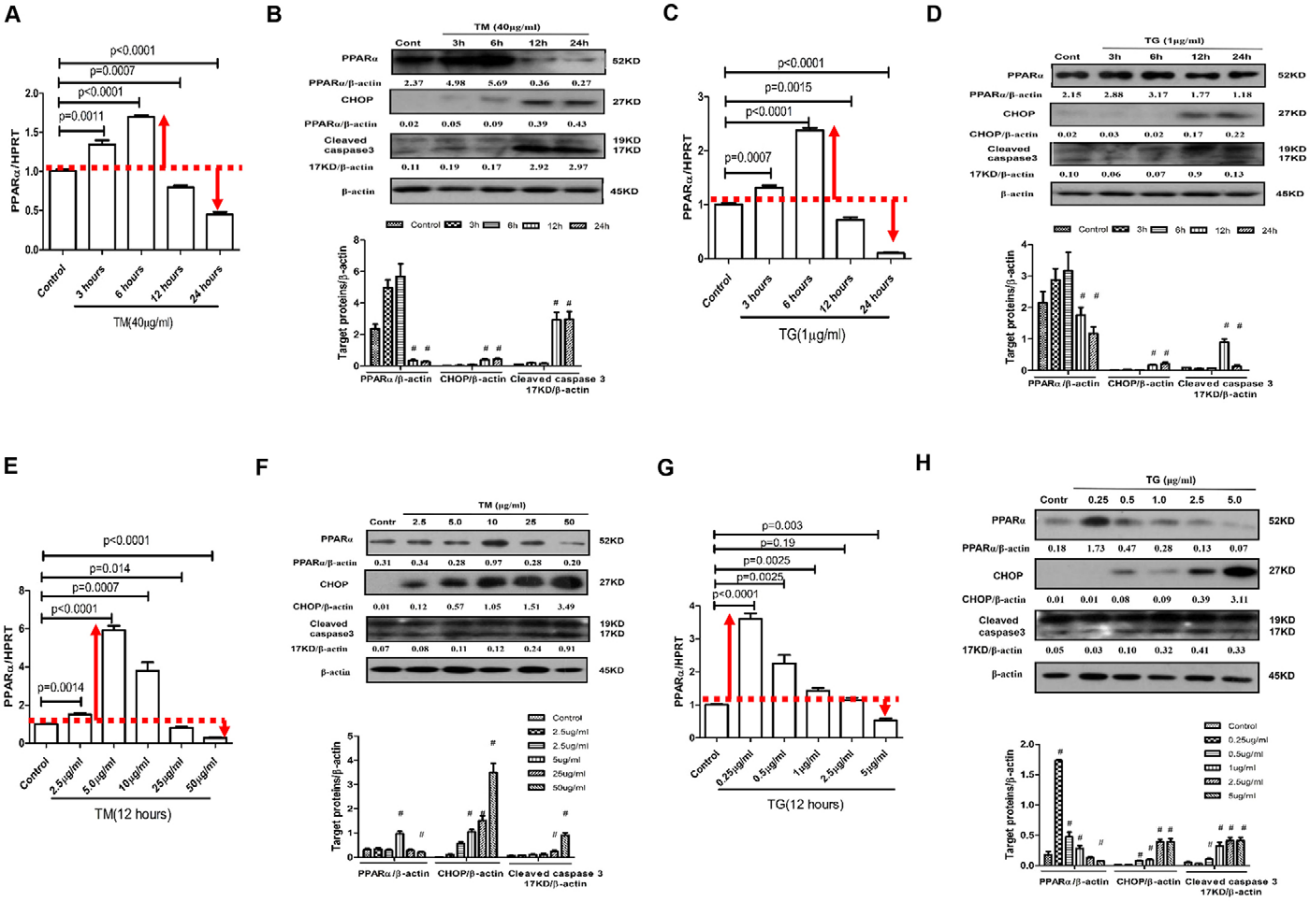
**Expression of PPARα is increased by mild ER stress and decreased by severe ER stress.** Primary hepatocytes were incubated with known ER stress inducers TM and TG for various times or at increasing doses. Cells in the increasing time group were treated with only PBS as a control or 40 μg/ml TM or 1 μg/ml TG for 3, 6, 12 or 24 h. Cells in the increasing concentration group were treated with 0, 2.5, 5, 10, 25 or 50 μg/ml of TM, or 0, 0.25, 0.5, 1, 2.5 or 5 μg/ml of TG for 12 h. (A,C,E,G) Relative PPARα mRNA expression was measured by qRT-PCR. (B,D,F,H) Protein levels of PPARα, CHOP, cleaved caspase-3 and β-actin were measured by western blotting. A representative blot from three independent experiments is shown. Densitometry analysis of protein levels was performed for each sample (compared with Control group, ^#^*P*<0.05). Data in A,C,E,G represented as means±s.d.

We further examined how PPARα is regulated in the progression of ER stress-induced primary hepatocyte apoptosis *in vitro*. The qRT-PCR and western blot results showed that the expression of PPARα was significantly upregulated in the early stage of tunicamycin (TM)- or thapsigargin (TG)-induced ER stress and was significantly downregulated in the later time points of TM or TG treatment compared with the control group (Fig. 5A-D). Moreover, there was a difference in responses at different doses of TM or TG, compared with the control group. A low dose of TM or TG markedly upregulated PPARα expression, whereas a high dose of TM or TG reduced the expression of PPARα (Fig. 5E-H). Moreover, in treatments with longer exposure and higher doses of TM or TG, cleavage of caspase-3 was increased (Fig. 5B,D,F,H). Therefore, these results showed that mild ER stress promotes the expression of PPARα, and severe ER stress reduces the expression of PPARα.

### The effect of PPARα regulation on ER stress-induced primary hepatocyte apoptosis *in vitro*

**Fig. 6.**
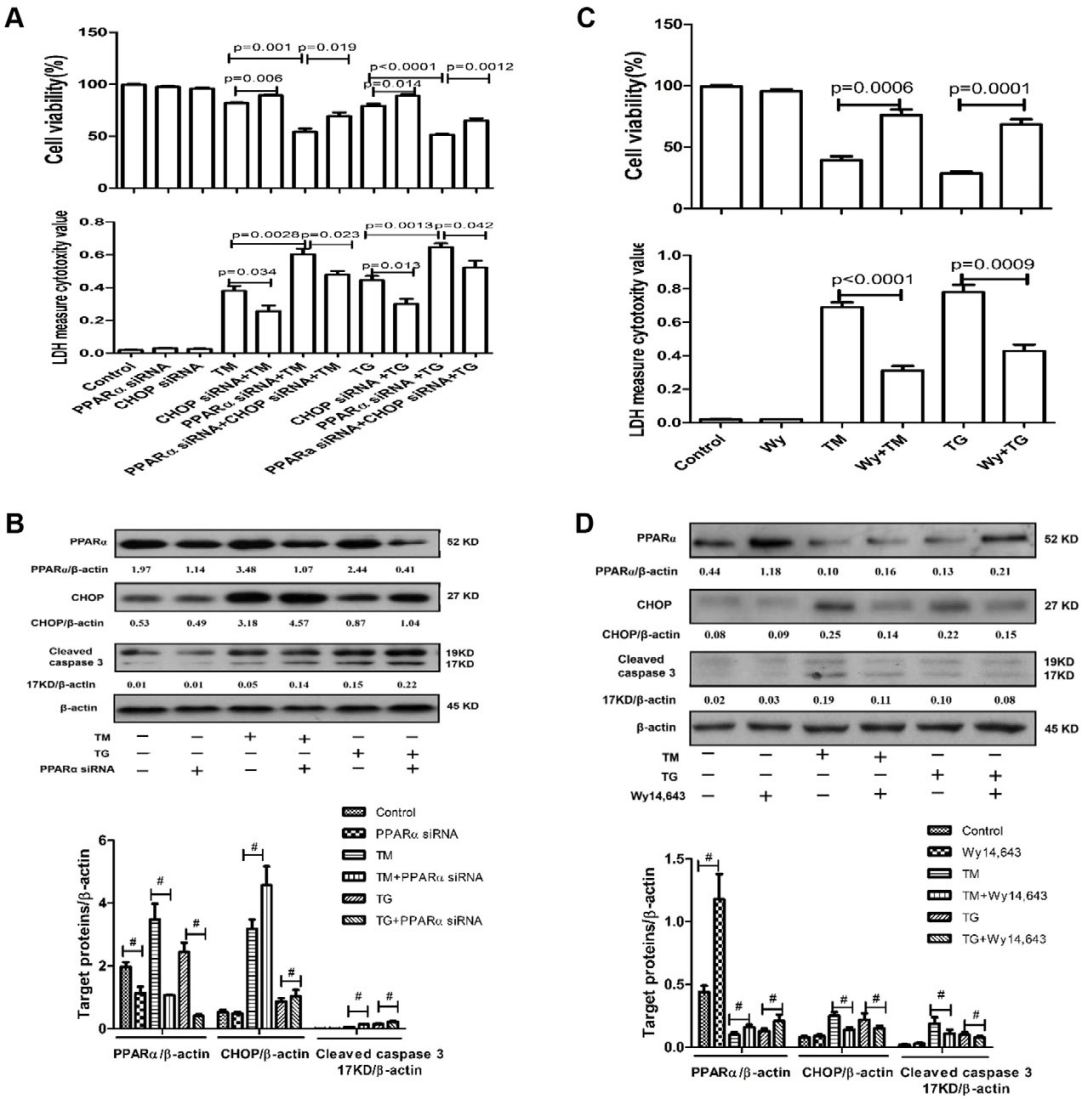
**PPARα can regulate ER stress-induced cell apoptosis *in vitro*.** (A,B) Primary hepatocytes were transfected with PPARα siRNA (5 nM), or control siRNA (5 nM), and/or CHOP siRNA (5 nM) for 24 h, followed by TM (40 μg/ml) or TG (1 μg/ml) for 6 h. Control cells were treated with only PBS. Cell viability or apoptosis was measured by MTT assay or LDH activity assay, respectively, separately in different groups. Protein levels of PPARα, CHOP, cleaved caspase-3 and β-actin in the different treatment groups were measured by western blotting. A representative blot from three independent experiments is shown. Densitometry analysis of protein levels was performed for each sample (^#^*P*<0.05). (C,D) Primary hepatocytes were incubated with Wy-14643 (50 μM) or DMSO for 2 h and then stimulated with TM (40 μg/ml) or TG (1 μg/ml) for 24 h. Cell viability or apoptosis was measured by MTT assay or LDH activity assay, respectively, separately in different groups. Protein levels of PPARα, CHOP, cleaved caspase-3 and β-actin in the different treatment groups were measured by western blotting. A representative blot from three independent experiments is shown. Densitometry analysis of protein levels was performed for each sample (^#^*P*<0.05). Data represented as means±s.d.

PPARα had been shown to be differentially regulated in the progression of ER stress. Therefore, we further analyzed the impact of PPARα on the intrinsic potential of primary hepatocyte apoptosis triggered by ER stress *in vitro*. Under conditions of mild ER stress, we used speciﬁc siRNA to knock down the expression of PPARα. TM or TG treatment for 6 h increased the release of lactate dehydrogenase (LDH) from the hepatocytes and decreased hepatocyte viability; downregulation of PPARα by siRNA further increased the LDH levels from the hepatocytes and further decreased hepatocyte viability (Fig. 6A). To evaluate the role of CHOP in the ER stress-PPARα pathway, we used siRNA to knock down CHOP and analyzed levels of MTT and LDH release in the different groups. The results indicated that, compared with the combination of PPARα siRNA and TM or TG treatment, the silencing of CHOP with siRNA partially improved cell viability and reversed the increases in LDH levels (Fig. 6A). Western blot analysis revealed that PPARα siRNA increased levels of CHOP and cleaved caspase-3 compared with TM- or TG-treated cells (Fig. 6B). Under conditions of severe ER stress, we used Wy-14643 to activate PPARα. Compared with 24 h treatment of hepatocytes with TM or TG, activation of PPARα by Wy-14643 significantly decreased the hepatocyte levels of LDH and increased hepatocyte viability (Fig. 6C). Western blot analysis also indicated that Wy-14643 decreased the levels of CHOP and cleaved caspase-3, as compared with TM- or TG-treated cells (Fig. 6D). Therefore, the activation or expression of PPARα was a key point of balance between hepatocyte survival promoted by mild ER stress and hepatocyte apoptosis induced by severe ER stress.

### The expression of CHOP and PPARα in the liver of individuals with HBV-related ALF

**Fig. 7.**
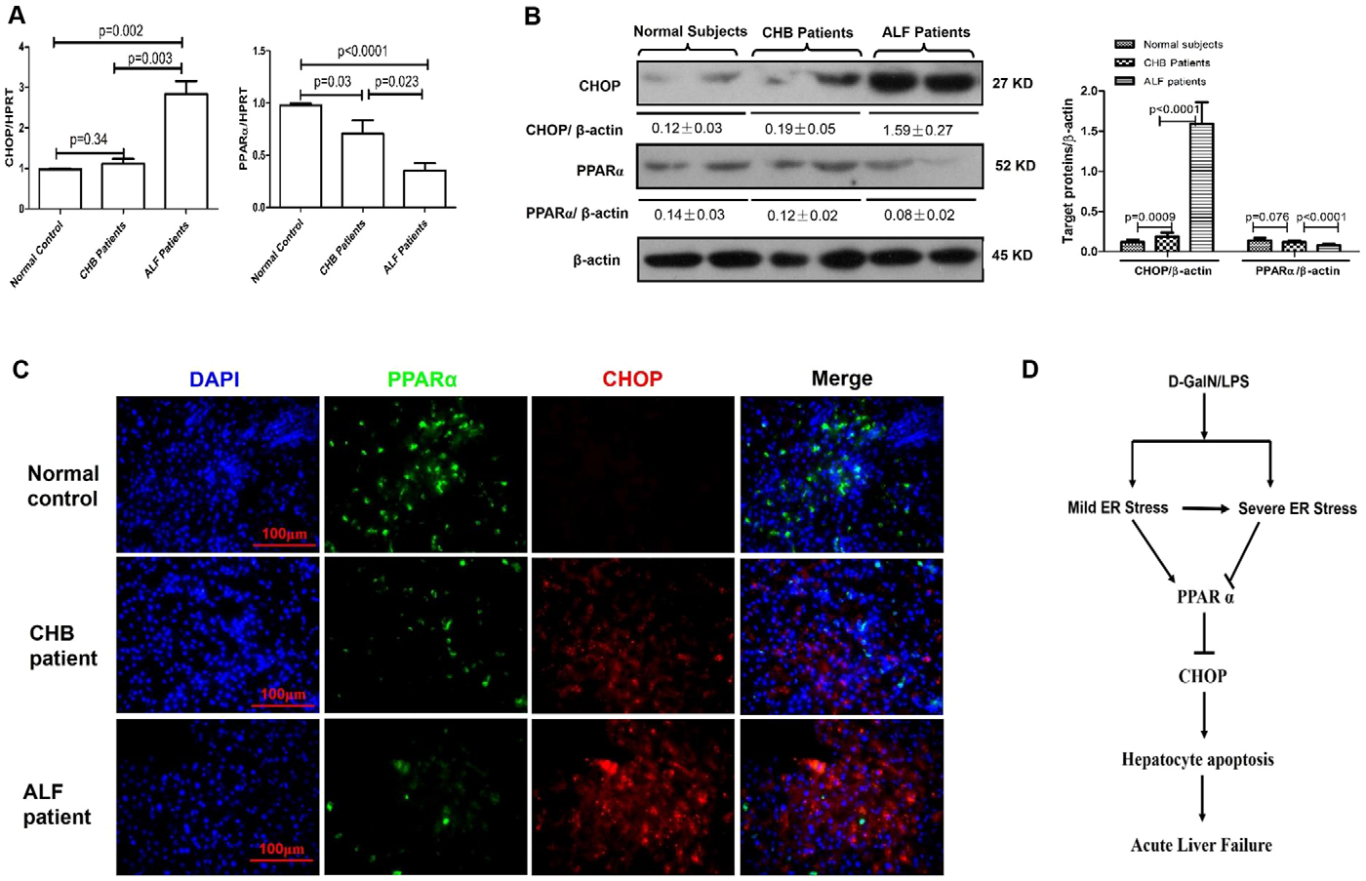
**PPARα is downregulated and CHOP is visibly increased in individuals with HBV-related ALF.** (A) Relative hepatic mRNA expression levels of PPARα and CHOP were measured by qRT-PCR in healthy controls (*n*=8), individuals with CHB (*n*=12) and individuals with ALF (*n*=12). (B) Protein levels of PPARα and CHOP were measured by western blotting. A representative blot from two samples of every group is shown. (C) Immunofluorescence staining for PPARα (green) and CHOP (red) in liver tissues from the patient groups. A representative experiment is shown. Original magnification 400×. (D) Schematic showing that in the progression of D-GalN/LPS-induced ALF in mice, mild ER stress is induced in the early phase of acute liver injury, which upregulates the expression of PPARα, but the severe ER stress is induced in the late phase of ALF, which downregulates the expression of PPARα. Decreased PPARα triggers CHOP activity, induces extensive hepatocyte apoptosis, and ultimately induces the development of ALF. Therefore, PPARα is a fulcrum in the regulation of ER stress-induced liver injury.

To investigate whether CHOP and PPARα are associated with the progression of ALF in individuals with HBV infection, we quantified the expression of CHOP and PPARα in liver tissues of control subjects, individuals with chronic hepatitis B (CHB) and individuals with HBV-related ALF. The qRT-PCR results revealed that CHOP gene expression increased significantly in individuals with ALF compared with the control subjects, but in the individuals with CHB, no significant changes were observed. PPARα gene expression gradually decreased in the progression of CHB to ALF (Fig. 7A); similar results were observed for protein levels by western blot analysis (Fig. 7B). Interestingly, the immunofluorescence staining revealed that the expression level of CHOP was low in hepatocytes in which PPARα was highly expressed (Fig. 7C). Thus, these results indicated that CHOP expression is upregulated, and PPARα expression is decreased in individuals with HBV-related ALF compared with healthy livers.

## DISCUSSION

In the present study, we demonstrate that PPARα activation significantly decreases hepatocellular apoptosis, thereby protecting mice from D-GalN/LPS-induced ALF. The protective mechanism of PPARα activation regulates ER stress and thus relieves liver injury caused by ALF in mice; moreover, PPARα could be a pivotal molecule that facilitates the transition from mild ER stress to progressively severe ER stress in ALF. Hence, the ER stress-PPARα pathway is necessary to the pathological mechanism of the ALF immune response cascade (depicted in Fig. 7D).

Acute liver failure (ALF) has a variety of etiologies including viral infection, acetaminophen damage, excessive alcohol intake, metabolic liver disease and other causes that remain unknown. It is associated with massive hepatocyte death through apoptosis or necrosis, but the precise mechanism is still not fully elucidated.Apoptosis, or programmed cell death (PCD), is actively induced by specific signaling cascades, including the intrinsic and extrinsic apoptosis signaling pathways, and occurs in a highly controlled fashion. Necrosis is viewed as a largely unregulated consequence of physicochemical stress characterized by mitochondrial impairment, depletion of adenosine triphosphate (ATP), and subsequent failure of ATP-dependent ion pumps. Recent evidence has indicated that PCD can also trigger a specific form of necrosis, termed necroptosis (Bantel and Schulze-Osthoff, 2012;Luedde et al., 2014). Elucidation of the regulated nature of multiple cell death modes not only furthers our understanding of the underlying pathophysiology but also suggests possible therapeutic treatment in diseases.

The first novel finding in this paper is that PPARα activation protects mice from liver injury by inhibiting ER stress-induced hepatocyte apoptosis in ALF. Prolonged or severe ER stress triggers cell apoptosis and several mediators of apoptosis are associated with ER stress-induced cell death. Some of the mediators are linked to the UPR sensors, but others are implicated in calcium and redox homeostasis. The transcription factor CHOP functions as the most well-characterized pro-apoptotic regulator.Previous studies have demonstrated that CHOP is significantly upregulated in GalN/LPS-induced ALF and is crucial in mediating ER stress-induced apoptosis (Rao et al., 2015), whereas silencing of CHOP reduces hepatocyte apoptosis in alcohol-induced liver disease (Ji et al., 2005; Tamaki et al., 2008). Our previous research has also shown that the expression levels of Grp78, Grp94 and CHOP are increased significantly in D-GalN/LPS-induced ALF, demonstrating the crucial role of ER stress-mediated hepatocyte apoptosis in the mechanisms of ALF (Chen et al., 2012). The studies have shown that PPARα plays a complex role in cell apoptosis. For example, PPARα shows duality in liver cancer; low amounts of PPARα activation increase cell apoptosis by changing the tumor microenvironment, and continued high levels of PPARα activation promote the growth of hepatoma carcinoma cells (Kimura et al., 2012). For normal cells, such as hepatocytes**,** vascular smooth muscle cells or kidney cells, PPARα activation suppresses apoptosis induced by various stimuli (Chung et al., 2012; Chen et al., 2013; Karakan et al., 2013). In the present study, we demonstrated that PPARα activation, through its agonist Wy-14643, downregulated expression of Grp78, Grp94 and CHOP and reduced D-GalN/LPS-induced ER stress-mediated cell apoptosis. Moreover, our results *in vitro* also indicated that knockdown of PPARα by siRNA or activation of PPARα by Wy-14643 promoted or inhibited ER stress-induced hepatocyte apoptosis, respectively. Furthermore, inhibition of ER stress directly upregulated the expression of PPARα in the ALF mouse model, and knockdown of PPARα reversed the protective effect of ER stress inhibition in the ALF mouse model. Together with previous findings, the results reported here support a mechanism whereby severe ER stress promotes the progression of D-GalN/LPS-induced ALF in mice by decreasing PPARα activation.

Another novel finding in this paper is that PPARα acts as a switch from mild ER stress to severe ER stress. ER stress and UPR have been linked to the pathophysiology of liver diseases. However, the UPR signaling pathways also play a crucial role in restoring ER homeostasis via PERK, IRE1 and ATF6. One set of effectors regulated by the UPR activates three adaptive signaling cascades to amoliorate ER stress. These adaptive mechanisms involve global attenuation of mRNA translation, which reduces the ER workload by blocking synthesis of new proteins; the upregulation of molecular chaperones, which expands the protein folding capacity of the ER; and the increase in ER-associated protein degradation (ERAD), which removes misfolded proteins from the ER (Treglia et al., 2012). Under sustained or massive ER stress, the UPR switches from an adaptive program to a pro-apoptotic program. CHOP protein is thought to be a crucial mediator of ER stress-associated apoptosis (Kim et al., 2008). Therefore, UPR activation elicits adaptive and pro-apoptotic effectors, and UPR signaling serves as a binary switch between adaptation and death. What are the molecular mechanisms to govern this transition? Chan et al. (2015) have shown that JNK functions as a key factor that regulates β-cell fate. In this paper, our findings suggest that PPARα could be a pivotal molecule that facilitates the transition from mild ER stress-induced cell survival to progressively severe ER stress-induced cell apoptosis. Our research has found that PPARα is expressed in normal hepatocytes and that mild ER stress upregulates the expression of PPARα, whereas severe ER stress downregulates the expression of PPARα. Knockdown of PPARα decreases mild ER stress-promoted hepatocyte survival, whereas the activation of PPARα decreases severe ER stress-induced hepatocyte apoptosis. Therefore, we believe that PPARα is a newly described mediator involved in the balance between adaptive and apoptotic factors regulated by the UPR.

In conclusion, we found that PPARα protects against ALF by suppressing ER stress-induced hepatocyte apoptosis. PPARα might be useful as a potential therapeutic agent to attenuate ALF. Further preclinical studies targeting PPARα agonists are warranted for the development of a clinically applicable treatment strategy to treat ALF.

## MATERIALS AND METHODS

### Animal experiments

Male C57BL/6 mice at the age of 8-12 weeks were purchased from the Capital Medical University (Beijing, China) and fed freely with a standard chow diet and water; they were housed under specific pathogen-free conditions for 1 week before the experiments. All animals received humane care according to the Capital Medical University Animal Care Committee guidelines.

The mice were intraperitoneally injected with D-GalN (700 mg/kg; Sigma, St. Louis, MO, USA) and LPS (10 μg/kg; InvivoGen, San Diego, CA, USA) to induce ALF, or with saline in the control animals. The PPARα activator Wy-14643 (6 mg/kg; Sigma) was administered via injection into the tail vein 2 h prior to D-GalN/LPS exposure. The downregulation of PPARα and CHOP were achieved by tail vein injection of specific siRNA (50 μM/kg; Jima, Suzhou, China). A chemical chaperone that relieves ER stress, 4-PBA (100 mg/kg; Sigma), was dissolved in PBS and administered intraperitoneally 6 h prior to D-GalN/LPS exposure. The mice were euthanized at 6 h after D-GalN/LPS treatment, and liver and serum samples were collected for future analysis.

### Human specimens

Control liver samples were collected from eight individuals undergoing hepatic resection for liver transplantation. CHB samples were obtained from the livers of 12 individuals undergoing liver puncture biopsy. ALF liver samples were obtained from the livers of 12 individuals with HBV infection undergoing liver transplantation, caused by acute exacerbation of chronic hepatitis B. This study was conducted in compliance with the 1975 Declaration of Helsinki, and the study protocol was approved by the Medical Ethics Committee of the Beijing YouAn Hospital. Written informed consent was obtained from all individuals or their families prior to enrollment. The clinical characteristics and details of the individuals included in the study are shown in the Table S1.

### Liver function tests and liver histological examination

Liver injury was estimated by biochemical serum markers such as albumin (ALB), ALT, AST, TBIL, ALP and by coagulation index such as PT and by pathological examination. Blood biochemical indicators were measured by using a multi-parametric analyzer (AU 5400, Olympus, Japan), according to an automated procedure. PT was detected using fully Automatic Coagulometer (Ac.T 5diff AL, Beckman-Coulter Inc., Brea, CA, USA). Liver tissue was fixed with 10% neutral formaldehyde and then embedded in paraffin. The specimens were cut into 5 μm sections, which were then stained with hematoxylin and eosin (H&E) and observed under light microscopy.

### Quantitative real-time polymerase chain reaction

Total RNA was isolated from 50 mg of liver tissue with TRIzol reagent, following the manufacturer's protocol. The RNA was reverse transcribed into cDNA using the SuperScript III First-Strand Synthesis System (Invitrogen, Carlsbad, CA, USA). Quantitative-PCR was performed using the DNA Engine with Chromo 4 Detector (MJ Research, Waltham, MA, USA). The reactions were set up in 20 μl total volumes with 1× SuperMix (Platinum SYBR Green qPCR Kit; Invitrogen), cDNA (2 μl) and 0.5 μM of each primer. The PCR cycle was as follows: 50°C for 2 min and 95°C for 5 min, followed by 50 cycles of 95°C for 15 s and 60°C for 30 s. The relative mRNA levels were normalized to the level of hypoxanthine-guanine phosphoribosyltransferase (HPRT) and calculated by using the 2^−ΔΔCt^ method. All samples were run in duplicate to ensure amplification integrity.

### Western blot analyses

Liver tissue samples were lysed in radio immunoprecipitation assay (RIPA) buffer containing phosphatase and protease inhibitors. After heat denaturation at 95°C for 5 min, proteins in SDS-loading buffer were subjected to electrophoresis in an SDS-12% polyacrylamide gel and subsequently transferred onto a PVDF membrane (Bio-Rad, Hercules, CA, USA). Primary antibodies against PPARα (1:1000; ab8934, Abcam, Cambridge, MA, USA), and Grp78 (1:1000; 3177), Grp94 (1:1000; 20292), CHOP (1:1000; 5554), caspase-3 (1:500; 9662), cleaved caspase-3 (1:500; 9664) and β-actin (1:1000; 4970), all from Cell Signaling Technology Inc., Santa Cruz, CA, USA, were used. The membranes were incubated with primary antibodies in TBST with 5% skim milk at 4°C overnight. Next, the membranes were washed with TBST three times and then incubated with horseradish peroxidase-conjugated secondary antibody (1:2000; 7074, Cell Signaling Technology) at room temperature for 1 h. The bands were visualized with SuperSignal West Pico chemiluminescent substrate (Thermo Fisher Scientific, Rockford, IL, USA) and developed by exposure on an X-ray film.

### TUNEL assay

Apoptosis in liver sections was detected by terminal deoxynucleotidyl transferase-mediated dUTP nick-end labeling (TUNEL, red fluorescence) using the In Situ Cell Death Detection Kit (Roche, Indianapolis, IN, USA). Negative controls were prepared through omission of the terminal transferase. Positive controls were generated by treatment with DNase. Nuclei were stained with 4′,6-diamidino-2-phenylindole (DAPI; 1 μg/ml; Shizebio, Shanghai, China) for 10 min. Images were obtained on a Nikon Eclipse E800 fluorescent microscope (Nikon Corp., Tokyo, Japan). After four fields were randomly selected from each section, 100 cells were successively counted for each field by a blinded observer and the ratio of TUNEL-positive cell number to the total cell number was calculated.

### Isolation and treatment of primary mouse hepatocytes

The livers of 7-week-old mice were perfused with collagenase-containing Hank's solution, and viable hepatocytes were isolated by Percoll isodensity centrifugation as described (Klaunig et al., 1981). To study the effects of PPARα regulation on hepatocyte apoptosis induced by ER stress, the cells were treated with TM (10 μg/ml; Sigma) or TG (1 μg/ml; Sigma), which increases ER stress, plus co-treatment with Wy-14643 (50 μM), and/or PPARα siRNA (5 nM), and/or CHOP siRNA (5 nM). The MTT assay (Amersco, Solon, OH, USA) was used as a qualitative index of cell proliferation. Hepatocyte apoptosis was evaluated by western blotting for cleaved caspase-3 and by the LDH assay (Biochain Institute, Hayward, CA, USA) of culture supernatants. The processing was conducted according to the manufacturer's instructions.

### Immunofluorescence staining

Paraffin sections were treated with xylene for 10 min three times. The sections were hydrated through a graded alcohol series and then rinsed three times with distilled water. After the sections were blocked for 20 min in 10% goat serum in PBS, they were incubated overnight at 4°C with the PPARα-specific rabbit polyclonal antibody (1:1000; ab8934, Abcam) and the CHOP-specific mouse monoclonal antibody (1:1000; 2895, Cell Signaling Technology Inc.). The slides were then incubated with Alexa Fluor^®^ 488 goat anti-rabbit IgG or Alexa Fluor^®^ 568 goat anti-mouse IgG (1:200; A-11034 and A-11031, respectively, Invitrogen) for 45 min. After three washes with PBS, the nuclei were stained with DAPI (1 μg/ml; Shizebio) for 10 min. The images were examined on a Nikon Eclipse E800 fluorescent microscope.

### Statistical analyses

The results are expressed as the means±standard deviation (s.d.). Statistical analyses were performed using unpaired Student's *t*-test or single-factor analysis of variance (ANOVA), and a value of *P*<0.05 (two-tailed) was considered significant.
